# Does welfare technology contribute to security, activity, participation and independence within municipal elder care? A qualitative study protocol

**DOI:** 10.1136/bmjopen-2024-094424

**Published:** 2025-02-03

**Authors:** Katarina Baudin, Johannes Österholm, Ingrid Hellström, Vedrana B Baric, Åsa Larsson Ranada

**Affiliations:** 1 Department of Neurobiology, Care Sciences and Society, Karolinska Institutet, Stockholm, Sweden; 2 Department of Health, Medicine and Caring Sciences, Division of Prevention, Rehabilitation and Community Medicine, Unit of Occupational Therapy, Linköping University, Linkoping, Sweden; 3 Department of Health Care Sciences, Marie Cederschiöld University, Stockholm, Sweden

**Keywords:** Aged, Digital Technology, Person-Centered Care

## Abstract

**Abstract:**

**Introduction:**

In Swedish municipal elder care, there is a growing expectation that welfare technology (WT) will play a pivotal role in addressing the increasingly complex needs of older persons who receive municipal elder care. As the ageing population continues to rise, the strain on welfare resources both, human and financial, intensifies. The adoption of WT holds promise for enhancing the well-being of older persons, their significant others, organisations and caregiving staff. However, the perspectives of older persons, significant others, staff and decision-makers’ on the aspects of security, activity, independence and participation that WT aims to influence needs further investigation. Thus, the overall purpose of the project is to explore how WT is perceived, implemented and experienced by different stakeholders within municipal elder care and to determine if, and how, WT contributes to the person’s security, activity, participation and independence.

**Methods and analysis:**

The project has a qualitative approach and will use thematic analysis. Data will be collected through semistructured interviews with different stakeholders—(a) older persons, (b) significant others, (c) staff and (d) decision-makers (ie, local politicians and local governmental officials)—in organisations within municipal elder care that use WT.

**Ethics and dissemination:**

The study is approved by the Swedish Ethical Review Authority (Dnr 2023-07203-01). All participants will provide informed consent. Dissemination of the results will be achieved through scientific publications and presentations at national and international conferences. During manuscript preparation, the Standards for Reporting Qualitative Research will be followed. The knowledge gained from the project will be shared with participating organisations, where presentations and discussions regarding the project will be offered. Stakeholders and the public will also be offered opportunities to attend seminars and lectures provided by participating universities.

**STRENGTHS AND LIMITATIONS OF THIS STUDY**

- This project employs a user perspective regarding the benefits of welfare technology from different stakeholders’ perspective; older persons, significant others, staff and decision-makers.
- Qualitative research methods (ie, semistructured interviews) will be used to elicit detailed accounts of the perspectives of different stakeholders regarding perceptions of welfare technology.
- Recruiting participants in municipalities with different prerequisites, such as demography, number of inhabitants, economic conditions and number of older persons, will contribute to a variety of conditions and experiences.
- Limitations of this study include potential sampling biases, as data are context-dependent, which can cause difficulties with transferability of the findings.

## Introduction

 Welfare technology (WT), a concept mostly used in a Nordic context, is defined as ‘all technology which in one way or another improves the lives of those who need it. The technology is used to maintain or increase security, activity, participation or independence for people with a disability or the elderly’.^1^ WT is thus expected to help older people extend their independence and hence improve their health. Furthermore, WT is often described as a possible solution to meet the challenges expected to arise within elder care due to demographic challenges (ie, an increased number of older people in need of care and, at the same time, fewer people of working age who can perform or finance this care).^2^,^5^ With the development of smart technologies and artificial intelligence, people’s use of digital technologies will continue to create new practical solutions for elder care. New forms of organisation and communication for public, private and social activities will characterise all sectors of society, not least health and elder care.^6^ There are great expectations that the implementation of WT in health and social care will result in a more efficient organisation of services, save resources, improve health and increase opportunities for social inclusion. Examples of this include facilitating ageing in place,^7 8^ monitoring symptoms^9^ and supporting self-care.^10^ Furthermore, WT is considered to have the potential to improve the staff’s work environment and the quality of the user’s care.^11^

At the same time, there is concern about how older people will be able to keep up with developments and take advantage of the opportunities provided by WT. The ability to use different WTs affects the perceptions of the technology and its relevance for the individual. With increasing age, several functional limitations appear due to natural ageing. These limitations, such as poor vision, hearing and fine motor ability, or a more fragile skin with reduced elasticity affecting the handling of touch technologies, might hinder technology use. This affects both the possibility of using the technology properly and the perception of the technologies.^8^ The development also places a demand on staff to handle new technology both practically and ethically and to keep up with its development. Exploring the perspectives of older people, significant others, staff and decision-makers (ie, local politicians and local government officials) on the use of WT is necessary to understand how WT can benefit all groups.^12^

Research in the area is mainly performed from the perspective of the technology, with the effect of various products being the main goal of the research, rather than what benefits the users or originates from the needs of the end users.^11 13 14^ Furthermore, there are various examples of projects where different WT have been implemented in different municipalities, but at the end of the project, the WT used is discontinued.^15 16^ Because of this, the knowledge within the area becomes fragmented when different organisations test various technologies but do not continue to use them or investigate the effect of the intervention in a more structured way. This also contributes to difficulties in deciding what technologies to invest in for the municipalities.^17^ Therefore, more knowledge is needed about how the use of WT is affected by the expectations and beliefs of those who use the technology in everyday life: the older persons, their significant others and the nearest care staff and how this permeates the organisations’ during the introduction. Thus, this project’s contribution will be to fill knowledge gaps in the field: how the end users within municipal elder care (older persons, significant others, staff and decision-makers) perceive and experience the implementation and use of WT as a broader phenomenon embracing various technologies.

### Theoretical positioning

The gerontological ecological theory of Lawton and Nahemow^18^ provides a framework for explaining how the interaction between the environment (ie, WT) and the person influences the individual′s behaviour and activity performance, including any functional limitations. It is commonly used in accessibility research related to older people.^19 20^ The concept of adaptation, based on Lawton’s and Nahemow’s theory, is used to explain that after a certain period of environmental exposure (ie, using the technology), the person gradually adapts so that the exposure is no longer felt or is perceived as neutral (they can manage the technology). The person has then achieved a level of adaptation where the environment is perceived as neither strong nor weak. Based on the theory, the competence–press model will be used to explain how behavioural outcomes occur and vary as a function of personal competence (ie, functional limitations) and environmental pressure (difficulty in using the WT). This model emphasises that there is a tension between a person’s capabilities and the demands of the surroundings. This means that a person with low functional capacity is more sensitive to environmental requirements and changes.^18^ Using this theory, and especially the competence–press model, might help to understand the impact and importance of WT on the different stakeholders and how it is domesticated by the different stakeholders.

### Purpose and research questions

The overall purpose of the project is to explore how WT—as a broader phenomenon embracing various technologies—is perceived, implemented and experienced by different stakeholders within municipal elder care, and how, and if, WT contributes to the individual’s security, activity, participation and independence.

By interviewing different stakeholders, that is, older people, significant others, staff and decision-makers (local politicians and local governmental officials) regarding their respective perceptions, expectations and implementation of WT, the following *research questions* will be addressed:

- In which parts of elder care is WT used, and for what purpose?
- How is the introduction of WT in elder care perceived by different stakeholders?
- What ideas and expectations about WT’s intentions to meet needs in elder care exist among various stakeholders?
- How is WT used in practice and how does this use correspond with the perceptions that exist for the introduction of WT?

## Methods and analysis

The project has a qualitative approach and will use reflexive thematic analysis,^21 22^ which is widely used within qualitative research to explore explicit and implicit meanings embedded within the data.

### Data collection

The intention is to carry out the project in different municipalities that use WT in their organisations of elder care. In Sweden, municipal elder care includes both home care and residential care facilities up to nurses’ level of healthcare. Older people in need of assistance in their daily life can apply for assistance from the municipality. The municipalities can provide the assistance within their own organisation or through private providers who have an agreement with the municipality regarding elder care.^23^

Organisations recruited to participate will be strategically selected to secure variation in municipal data, with different prerequisites that might influence the implementation of WT, such as demography, size, economic conditions, number of older persons, etc. In each participating organisation, we will try to involve all stakeholder groups, if possible.

The study period commenced in June 2023 with data collection ongoing until January 2025. The project will be finally reported to the funder in June 2027.

Data will be collected through semistructured interviews with different stakeholders—(a) older persons, (b) significant others, (c) staff and (d) decision-makers, in organisations within municipal elder care that use WT. Individual semistructured interviews will be conducted with these different groups of stakeholders to highlight the user’s perspective from their perspectives. The intention is to interview 15 participants from each group of stakeholders, giving a total of 60 interviews. A semistructured interview guide^24^ based on previous research^11 13 14^ focusing on the experiences, perceptions and domestication of WT will be used in the interviews (specified in relation to each group of stakeholders) (see online supplemental file 1). The use of semistructured interviews in this project offers the flexibility to alter questions in response to interviewees’ answers, allowing researchers to pose follow-up questions in complement to the prespecified themes or areas and to the prepared questions.^24^ The interviews will be audio-recorded and transcribed verbatim for analysis.

Interviews with older participants will be conducted in person, while interviews with other participants may be done in person or digitally through video call. In the case of digital interviews, only audio will be recorded.

### Inclusion of participants

A strategic selection will be used, where individuals with relevant experience in using or making strategic decisions regarding WT within elder care will be recruited as participants for the project. As this project utilises WT as a broader phenomenon encompassing various technologies, any type of WT used within the municipality will be eligible for inclusion. To reach informants, contact will be made with operational managers or equivalents for help in asking staff, older persons and significant others. Municipal family centres will also be contacted for the recruitment of significant others. Decision-makers will be approached through official channels (ie, homepages of organisations, etc).

To be included as an older participant, the person needs to use WT and be a user of municipal elder care. The individual must be over 65 years of age.

For significant others to be included, they need to have a relationship with a person who meets the above criteria.

For staff to be included, they need to work with any sort of WT within municipal elder care and have at least 1 year of experience in this field.

Decision-makers (ie, local politicians and local government officials) will be included if their role or job involves responsibility for and making strategic decisions about technology in elder care.

In the research group, well-established networks will be utilised to get in contact with potential participants to recruit. Additionally, new contacts through official contact channels will be established to ensure a variety of collected data.

### Patient and public involvement

An advisory board with the purpose of critically review and contributing to the development of the project will be recruited. The advisory board shall consist of people with interest, knowledge and experience in the areas covered by the research questions, such as experts in the fields of WT, elder care, qualitative research, etc, and representatives from participating organisations. Representatives from pensioners’ and patient organisations (such as PRO, Dementia, Stroke) will also be asked to participate to ensure relevance for end users.

### Data analysis

The project design provides four distinct data sets from different stakeholders’ perspective on the studied phenomenon. These data sets will be used to explore all four research questions from each stakeholders’ perspective (individually) in separate publications. To explore these research questions from multiple stakeholders’ perspective, an additional article exploring the shared meaning of the studied phenomenon will use all four data sets.

The data will be analysed using the reflexive thematic analysis method.^21 22^ This method is applicable as it permits analysing data both inductively and deductively. The analysis follows several phases, including reading the transcribed material repeatedly to gain a deeper understanding of the content. Data of relevance (data items) for the study’s aim is then extracted, which builds up the data set^21^ and is used in the data analysis. The data items are then condensed to retain their principal content and labelled with a code. These condensations and codes are then used to create ‘stories about specific patterns of shared meaning within and across the data sets’ (22, p592). Reflexive thematic analysis is designed to develop a more nuanced reading of the data and is implemented with theoretical assumptions and philosophical sensibility.^25^ Each stakeholder’s perspective will be processed, as will the various research questions from the perspective of two or more stakeholders, depending on what is relevant based on each question; for example, questions connected to the implementation of WT in the organisation are more relevant to staff and decision-makers and will focus on these stakeholders’ perspectives. When appropriate, the results of the analyses will be discussed in relation to the different parts of the competence–press model.^18^

### Study trustworthiness

To strengthen the trustworthiness of the findings, different strategies will be employed, as suggested in the qualitative research literature.^26^ Credibility will be attained by involving different authors independently in the different steps of the analysis, as suggested by Braun and Clark.^21^ Reflexive practices, such as an audit trail of decisions made during data collection and data analysis, will be used to enhance the dependability of the findings.^26^ Confirmability of the results will be addressed by providing a detailed description of participants and the context, with the intention of enabling the reader to assess the transferability of the results to other contexts.^26^ The participating researchers have backgrounds in various areas such as WT, social services, occupational therapy, nursing and clinical research involving older people, significant others, staff and decision-makers. The research group’s competence, with a broad knowledge in qualitative methods and the different stakeholders, contributes to the feasibility of the project.

Furthermore, to enhance the rigour of the study, the Standards for Reporting Qualitative Research^27^ will be used.

## Ethics and dissemination

The study is designed and will follow the ethical standards for scientific work based on the Declaration of Helsinki.^28^ The study is approved by the Swedish Ethical Review Authority (Dnr 2023-07203-01). Participants will be informed that their data will be used in research but coded and presented at group level, ensuring they will not be individually recognised. Citations will be labelled with stakeholder group and age to keep them pseudonymised.

At the initial appointment, the researcher will seek written or oral (recorded) informed consent, and, if appropriate, proceed to collect interview data. If it is established that an individual is unable to provide written consent because of literacy, vision or motor problems, verbal consent will be arranged. This procedure will also be used when interviews are conducted digitally.

Dissemination of the results will be achieved through scientific publications and presentations at national and international conferences. In addition, the results are expected to be presented in popular scientific journals in Sweden. Everyone in the research group is affiliated with professional education at the participating universities and colleges, where the results of the project can be directly used in teaching.

Contact has been established with several authorities that operate in the areas of elder care and WT, which will be utilised to disseminate the results. The knowledge gained from the project will also be shared with participating organisations, where presentations and discussions regarding the results will be offered. Stakeholders and the public will also be given opportunities to attend seminars and lectures provided by participating universities.

## Discussion

With more knowledge on why and how WT contributes to the aspects of security, activity, participation and independence, interventions in the field can be administered more efficiently.^13^ Knowledge is also needed about why different new innovative products are implemented in the organisations (ie, what are the intentions with a technology and what needs of the older person is targeted). Is it the organisation’s access and ability to introduce WT that governs this, or is it based on the existing needs of the individuals and/or staff in elder care? This question still needs to be illuminated in research from different aspects, both from the end users, that is, older persons, significant others and staff, and from the decision-maker’s perspective.^12^

A feasibility study in the field of WT^11^ showed that based on published research, it can be difficult to interpret whether WT contributes to security, activity, participation or independence, which shows that further research from a user perspective is needed. Previous studies have mostly focused on the effectiveness of one specific technology at a time,^6 10^ and studies are also underway that address the working conditions of staff in relation to WT.^6 29 30^ This project intends to fill knowledge gaps that still exist in the field: how the phenomenon of WT, comprising various technologies, is perceived, implemented and experienced by different stakeholders within municipal elder care (older persons, significant others, staff and decision-makers) and how, and if, WT contributes to the individual’s security, activity, participation, and independence.

## supplementary material

10.1136/bmjopen-2024-094424 online supplemental file 1

## References

[1] Nordic welfare center. (n.d.) https://nordicwelfare.org/en/welfare-policy/welfaretech/.

[2] World Health Organization (2015). World report on ageing and health.

[3] Socialstyrelsen (2020). Vård och omsorg om äldre. lägesrapport 2020. [in swedish: national board of health and welfare. care and care for older people. progress report].

[4] Sveriges Kommuner och Landsting (SKL) (2018). Ekonomirapport, maj 2018. [in swedish, swedish association of local authorities and regions. financial report.

[5] Nordic welfare center https://nordicwelfare.org/en/welfare-policy/.

[6] Lydahl D (2019). Välfärdteknikens värden – effektivvård och hållbart arbetsliv eller avlägsen vård och övervakade anställda. forteansökan 2019-01452.

[7] Elers P, Hunter I, Whiddett D (2018). User Requirements for Technology to Assist Aging in Place: Qualitative Study of Older People and Their Informal Support Networks. JMIR Mhealth Uhealth.

[8] Hunter I, Elers P, Lockhart C (2020). Issues Associated With the Management and Governance of Sensor Data and Information to Assist Aging in Place: Focus Group Study With Health Care Professionals. JMIR Mhealth Uhealth.

[9] Pajalic Z, de Sousa DA, Strøm BS (2023). Welfare technology interventions among older people living at home-A systematic review of RCT studies. PLOS Digit Health.

[10] Lee J, Yeom I, Chung ML (2022). Use of Mobile Apps for Self-care in People With Parkinson Disease: Systematic Review. JMIR Mhealth Uhealth.

[11] Myndigheten för delaktighet (2018). En Förstudie Inom Området Välfärdsteknik – Inför En Eventuell Kunskapsöversikt.

[12] Borg J, Gustafsson C, Landerdahl Stridsberg S (2023). Implementation of welfare technology: a state-of-the-art review of knowledge gaps and research needs. Disabil Rehabil Assist Technol.

[13] Zander V, Gustafsson C, Landerdahl Stridsberg S (2023). Implementation of welfare technology: a systematic review of barriers and facilitators. Disability and Rehabilitation: Assistive Technology.

[14] (2020). Vårdanalys innovation efter funktion. välfärdteknikens effekter ur fyra perspektiv [in swedish: care analysis. innovation by function. the effects of welfare technology from four perspectives].

[15] Frennert S (2020). Approaches to welfare technology in municipal eldercare. J Technol Hum Serv.

[16] Nordic Welfare center (2017). Weldfare technology tool box. https://nordicwelfare.org/wp-content/uploads/2017/10/england_webb.pdf.

[17] Socialstyrelsen (2024). E-hälsa och välfärdsteknik i kommunerna 2024 in swedish. national board of health and welfare. e-health and welfare technology in municipalities 2024. https://www.socialstyrelsen.se/globalassets/sharepoint-dokument/artikelkatalog/ovrigt/2024-5-9099.pdf.

[18] Lawton MP, Nahemow L, Eisdorfer C, Lawton M (1973). The Psychology of Adult Development and Ageing.

[19] Iwarsson S, Ståhl A (2003). Accessibility, usability and universal design--positioning and definition of concepts describing person-environment relationships. Disabil Rehabil.

[20] Iwarsson S, Nygren C, Oswald F (2006). Environmental Barriers and Housing Accessibility Problems Over a One-Year Period in Later Life in Three European Countries. J Hous Elderly.

[21] Braun V, Clarke V (2006). Using thematic analysis in psychology. Qual Res Psychol.

[22] Braun V, Clarke V (2022). Thematic analysis: a practical guide.

[23] Social Services Act (2001). Stockholm: ministry of social affairs.

[24] Brinkmann S, Kvale S (2015). InterViews: learning the craft of qualitative research interviewing.

[25] Braun V, Clarke V (2019). Reflecting on reflexive thematic analysis. Qual Res Sport Exerc Health.

[26] Patton M (2015). Qualitative Research & Evaluation Methods: Integrating Theory and Practice.

[27] O’Brien BC, Harris IB, Beckman TJ (2014). Standards for reporting qualitative research: a synthesis of recommendations. Acad Med.

[28] World Medical Association (2013). Declaration of helsinki. ethical principles for medical research involving human subjects. https://www.wma.net/policies-post/wma-declaration-of-helsinki-ethical-principles-for-medical-research-involving-human-subjects.

[29] Gustafsson C (2019). Faktorer som underlättar eller hindrar implementering av välfärdsteknik. forteansökan 2019-01515.

[30] Christensen J (2020). Kunskapsöverföring av välfärdsteknik – gränsöverskridande kommunal äldreomsorg. forteansökan 2020-01288.

