# Supplementary material for: Does welfare technology contribute to security, activity, participation and independence within municipal elder care? A qualitative study protocol
Source: BMJ Open. 2025 Feb 3;15(2):e094424. doi: 10.1136/bmjopen-2024-094424 (PMC11795365; doi:10.1136/bmjopen-2024-094424)
Supplement: online supplemental file 1 [file bmjopen-15-2-s001.docx]

**Supplement: Topic guides for interviews**

***Research questions that form the basis for the design of the interview guide***

1. In which parts of elder care is welfare technology used, and for what purpose?
2. How is the introduction of welfare technology in elder care perceived by different actors?
3. What ideas and expectations about welfare technology's intentions to meet needs in elder care exist amongst various actors?
4. How is welfare technology used in practice and how does this use correspond with the perceptions that exist for the introduction of welfare technology?

**Questions to be selected depending on informant (**older people, significant others, staff, and decision-makers)

**Demographic questions:** age, gender, education, employment/position, organisation,

**Question area *Use and Purpose of welfare technology* (research questions 1 & 4)**

Kind of welfare technology, expectations to achieve with this welfare technology, Plans for welfare technology going forward, What is this welfare technology for?

How does the use of this welfare technology affect your work? What kind of WT do you have as digital support for you as a staff member, Do you receive any knowledge support when introducing welfare technology at your company?

**Question area *Implementation/Introduction of welfare technology* (research question 2)**

How does it work when you introduce new welfare technology in your organization? Can you describe the work process, On what grounds is the introduction of welfare technology decided, How is this initiated? Can you describe how it works? Who is involved in this decision? What do you think about that?

What are the latest WT you have implemented in your municipality? What is your role in the implementation of welfare technology at your organization? Can you tell us about an example of a good implementation of welfare technology in an elderly patient/resident and how it was done? Can you tell us about an example of an implementation of welfare technology in an elderly patient/resident that did not work as well?

How does the decision-making process usually work when you buy welfare technology for your organization? Are you involved in this in any way?

**Question area *Preconceptions and Expectations (*research questions 3 & 4)**

How do you see that welfare technology does any good? Do you have any kind of evaluation/follow-up after the implementation? Do you usually do any kind of evaluation of the effects of welfare technology in your municipality? Are there any advantages of welfare technology – which ones? Are there any challenges with the implementation of welfare technology - which ones? Digitalization is advancing rapidly in our society today, as is advanced technology. How do you see this in relation to welfare technology? Do you think that something needs to be changed in your organization to meet this development? Do you think about the sustainability aspect when you bring new welfare technology into your organization? The circular economy? What is the structure of IT around welfare technology? How do technology updates/servicing work?

Welfare technology and good and close care are said to mean that the patient/user can increase their independence/autonomy, to make decisions about and take control of their everyday life. What do you think about that? How can it affect your work? How do you think about welfare technology and the personal integrity of the elderly? Can you give some/some examples? How can it affect your work? How is the information generated by welfare technology handled? How does it affect your work? Do you usually do any kind of evaluation of the effects of welfare technology in your municipality? How do you think about the future and welfare technology? Do you think it's fast or slow?

**Final Questions**

Based on your thoughts before and during this interview, is there anything I forgot to ask about or that you think I should have brought up? Is there anything you would like to ask me about?
