# Supplementary material for: Does welfare technology contribute to security, activity, participation and independence within municipal elder care? A qualitative study protocol
Source: BMJ Open. 2025 Feb 3;15(2):e094424. doi: 10.1136/bmjopen-2024-094424 (PMC11795365; doi:10.1136/bmjopen-2024-094424)
Supplement: Supplementary file 2 [file bmjopen-2024-094424.reviewer_comments.pdf]

PEER REVIEW HISTORY

BMJ Open publishes all reviews undertaken for accepted manuscripts. Reviewers are asked to complete a checklist review form and are provided with free text boxes to elaborate on their assessment. These free text comments are reproduced below.

ARTICLE DETAILS

Title (Provisional)

Does welfare technology contribute to security, activity, participation, and independence within elder care? - a qualitative study protocol

Authors

Baudin, Katarina; Österholm, Johannes; Hellström, Ingrid; B. Baric, Vedrana; Åsa, Larsson Ranada

---

VERSION 1 - REVIEW

Reviewer 1
Name Silies, Katharina
Affiliation University of Lübeck, Institute for Social Medicine and Epidemiology, Nursing Research Unit
Date 08-Nov-2024
COI I understand and consent to the named publication of this review.

Dear authors, I have been very pleased to have the opportunity to review your manuscript.

You present the protocol for a qualitative study on welfare technology in elder care, which is an important topic. I appreciate that you thus support transparent reporting and your manuscript is overall well structured and informative.

I have some minor suggestions to add some information in your methods section, please find my detailed review below.

Detailed review:

Major comments (content)

Title:

1. Please check grammar: it should be „Does welfare technology …“ or „Do welfare technologies…“ – anyway, I would like to suggest a slight modification (if it is not too long): Welfare technologies‘ contribution to safety, activity, participation, and independence in

elder care organisations – protocol for a qualitative study with older persons, significant others, staff and decision makers.

2. P3, L10: Strengths and limitations: I recommend not to abbreviate WT in this bullet point.

3. P3, L19: … a strategic selection – you might consider the term purposive sampling strategy?

Introduction/Background

4. P4, L31: I recommend to move the definition of WT to the top of the introduction.

5. P5, L44: ... the environment (i.e. WT) – I wonder, as technology is mentioned later in the sentence, whether the environment should be defined as the elderly care (facility) in this place?

Methods

6. Please consider some information on research team and reflexivity: what kind of professional background or research focus do the researchers have?

7. P7, 42: The range of your targeted sample size is very broad. How do you determine the final size? How will you decide, whether 10 or 20 participants of each group will be the better fit? Will you discuss the concept of data saturation? You have a very broad approach which might make it difficult to identify saturation. Therefore, you might want to specify it here.

8. P7, L43: … previous research: please add a reference.

9. Please also add the topic guides for your interviews as a supplement.

10. P8, L15: … using or making strategic decisions regarding WT …: please define, what kind of WT will be considered as inclusion criterion, and whether there are any exclusions in this regard. Please also define municipal elder care: is this inpatient / residential care or outpatient / home care, or both? Does municipal refer to the institution as being funded by municipality? Are privately funded institutions excluded? Maybe this concept does not even exist in Sweden, but it does in other countries, so an explanation to the international reader will be appreciated. Will you try to include all stakeholder groups within participating institutions, or will you recruit on the individual level?

11. P8, L44: … new contacts will be established: in which way?

12. P8, L44: … to ensure a variety of collected data: what is your aim with regard to variety? Does it refer to the technologies involved?

13. P8, L47f: advisory board: I assume that the advisory board is already in place, as you are almost done with data collection? Then I would recommend to give more detailed information (profession, organisation or sector, number of participants) on your advisory board

14. P9, L42f: “The collected material will be analysed based on the respective publications that are planned”: I should assume, that the research aim comes first and the publication second? I recommend to state the research aim and question for each participant group and the combined aim and question.

15. P9, L55: When appropriate … - This sounds slightly contradictive to what you state earlier as research questions and have in mind as final topics (safety etc, see your title). I am sure you will be easily able to clarify what your aim is, or with which methods you will analyse the different concepts you mention throughout your paper.

16. P10, L25f: Data statement section: You should describe here what your plan for data sharing is. It is also not about access of your authors and team to data but about access of independent researchers and transparency of your research. If you do not plan to make raw data (=transcripts in this case) accessible, you should state it here and give a reason. I really recommend to read: “Recommendations for the Conduct, Reporting, Editing, and Publication of Scholarly Work in Medical Journals” – this is a brilliant paper that summarizes about everything that is helpful when publishing. See part III, letter L, number 2 on data sharing.

17. P10, L45: … will be pseudonymized: in which way will participants receive a pseudonym and how will it be linked to personal data? Or could you also describe your method as anonymized?

Discussion

18. P11, L31: … safety, activity, …: How will you ensure, that these concepts will be explored in your research? Will you explicitly incorporate these in your interview guides / questions? Or will you use them as predefined concepts in your analysis? Then you should explain it in the methods section.

Minor comments (language, tables and figures, …)

19. A general remark: please consider to use first person active voice, this is recommended by many journals.

20. Overall your language is clear, there are some minor typos, please check your manuscript carefully before resubmission. I recommend to consider a native or C2-level English speaker to do a final check.

Good luck!

Katharina Silies

**Reviewer**	**2**
**Name**	**Elaswarapu, Rekha**

Affiliation **King's College London - Strand Campus, NIHR Health & Social Care Workforce Unit**

Date **10-Nov-2024**

COI **I understand and consent to the named publication of this review.**

This is an interesting topic relevant to care of the elderly. A couple of points to highlight are:

1. level of health related morbidity may influence perception of the use of WT;

2. Familiarity and comfort with the use of WT could be a factor to consider so someone who is a technophobe may start from an entirely different point of view in their perspective of the use of WT.

Reviewer **3**

Name **Elf, Marie**

Affiliation **Dalarna University, School of education, health and social studies**

Date **10-Nov-2024**

COI **I understand and consent to the named publication of this review.**

Comments on the manuscript “Do welfare technology contribute to safety, activity, participation, and independence within elder care? -a qualitative study protocol Journal” bmjopen-2024-094424

Thanks for the opportunity to read and comment on your manuscript about welfare technology (WT). As you write, welfare technology is common and will play a pivotal role in elder care today, but it’s not explored enough when it comes to how stakeholders perceive welfare technology and how it contributes to the individual's security, activity, participation, and independence. The manuscript is well written and easy to follow.

My main concern is that I cannot clearly identify your specific contribution with the study. While WT has been studied extensively, it’s important to highlight what makes your work unique or distinct within this broader field. Consider focusing on what new insights, methods, or perspectives you bring.

You have not explained what kind of WT you will explore. Since WT is a central theme, the introduction should define what is meant by welfare technology, especially in the context of elder care. Implicitly I can understand that you mean high tech but that must be clear. WT doesn't have to refer to high-tech devices. It can mean a wide range of tools, including non-digital or aids such as wheelchairs, walkers, and other equipment to support the well-being, independence, and quality of life of individuals who may have physical or cognitive disabilities.

While welfare technology often suggests digital innovations like health-monitoring apps, smart home devices, or telehealth platforms, the term can also apply to more traditional assistive devices that serve the same goals. You need to give examples of what kind of WT and devices you mean.

In addition, you need to write more clearly what WT can contribute with when it comes to improved service, work environment, health etc…in what way I keep asking when reading the introduction.

You also need to more clearly discuss the role of WT in addressing safety, activity, participation, and independence. For example, how can WT (which one) enhance the safety of older adults? How might WT (which one) support physical activity? What role does technology play in helping older adults remain socially connected or engaged with their communities? How does welfare technology facilitate independence in daily living?

Moreover, you write that the project can contribute knowledge on the implementation and consequences of WT for older persons, significant others, and staff, then the introduction should address implementation of WT and its challenges as a focus.

What do you mean by implementation in the context of WT in elder care? This might include discussing what aspects of implementation are relevant, such as the processes, challenges, and necessary conditions for successfully integrating technology. How can implementation science explain the effectiveness and usability of WT for older persons, as well as its acceptance by staff and family members. Do you think implementation success differ among groups (older persons, significant others, and staff)?

Without mentioned or explain further how WT can influence safety, independence etc… and implementation, the introduction feels incomplete or disconnected from the project's main objectives.

I wonder why your research questions do not mention safety, independence, participation and implementation? I cannot see the connection to the title.

You use safety and security interchangeable? Do you mean it is the same? For me they are slightly different concepts.

What questions will you ask the stakeholders? Can you give some examples?

In which context are you planning to do your research? In elder care in normal housing or at residential care facilities? In several municipalities or in one? Could older adults have any disabilities? And what kind of WT? We need more information.

Are you using any framework for reporting qualitative studies such as COREQ?

**Reviewer**	**4**
**Name**	**Potter, Kathleen**
**Affiliation**	**Ryman Healthcare Ltd**
**Date**	**11-Nov-2024**

COI I understand and consent to the named publication of this review.

This paper describes a study protocol for a qualitative investigation of the thoughts and feelings of four different groups about the use of welfare technologies in the care of older people who require municipal care. The paper is generally well structured, clear and contains an appropriate level of detail. The study subject is important and relevant and I look forward to reading the authors' findings in due course.

I have a few suggestions for improving the protocol description as noted below.

1. The paper would benefit from providing some more definitions of the terms used in a way that an international readership will understand. The terms that need definition early, including in the abstract preferably are:

a. Municipal elder care – this may be a specific European term or it may be a specific Swedish term, but it is not clear to this Australasian reader exactly what it means. Is it care provided in the home or is it care provided via an institution (a residential age care facility, for example) or is it both? Some clarification is required.

b. Welfare technology – this term is defined in the second paragraph of the introduction, but it needs to be defined in the first paragraph I believe, so that the reader knows exactly what is being studied. Similarly, there needs to be a brief definition provided in the abstract. Some examples would also be useful, to help the reader understand the scope of the technologies being considered in the research.

c. It would also be helpful to define how the researchers understand the terms 'security, activity, participation and independence" in the context of the demographic they are studying. A box might be useful, with all these terms defined, as the authors are applying them in their research, and this could be referred to by the reader, if the authors do not wish to include the definitions in the text of the paper.

2. This is a general comment, but the scope of the project seems very broad. On one had this is commendable, as the authors will capture a wide range of information about their subject, but on the other hand it does make me wonder how they will synthesize all this information into some coherent summary of the topic.

3. A little more detail about how the authors will ensure an unbiased sampling of the population they are interested in interviewing would be useful. Using existing research networks is acceptable, but there is a danger of reinforcing an existing echo-chamber with only similar voices being heard by this approach. I would like to see more details about the efforts the authors have made to ensure their recruitment methods reach voices who are not always represented in this type of research. Will they interview immigrant care staff for instance, or only native Swedes?

4. There are a few clunky bits of English in the paper and a couple of simple spelling/grammatical errors. The authors would benefit from asking a native English paper to read the draft and correct these minor errors. Two I picked up on were Page 7, line 3 ‘explains’ should be ‘explain’ and Page 7, line 8 ‘difficulty to use’ should be ‘difficulty in using’. There were several others.

Overall this is a great project and very timely and topical. The authors are to be commended on undertaking a challenging and interesting project and I look forward to reading their papers.

VERSION 1 - AUTHOR RESPONSE

Reviewer: 1
Dr. Katharina Silies, University of Lübeck
Comments to the Author:

Dear authors, I have been very pleased to have the opportunity to review your manuscript.<br>You present the protocol for a qualitative study on welfare technology in elder care, which is an important topic. I appreciate that you thus support transparent reporting and your manuscript is overall well structured and informative.	Thank you for your supporting comment.
I have some minor suggestions to add some information in your methods section, please find my detailed review below.<br>Title:<br>1. Please check grammar: it should be „Does welfare technology …“ or „Do welfare technologies…“ – anyway, I would like to suggest a slight modification (if it is not too long): Welfare technologies‘ contribution to safety, activity, participation, and independence in elder care organisations – protocol for a qualitative study with older persons, significant others, staff and decision makers.	This has been adjusted
2. P3, L10: Strengths and limitations: I recommend not to abbreviate WT in this bullet point.	This has been adjusted
3. P3, L19: … a strategic selection – you might consider the term purposive sampling strategy?	We have chosen to keep the strategic selection as this is what we are doing when considering different municipalities to ask to participate. We have strategically selected

	large, medium and small municipalities in different areas.
Introduction/Background<br>4. P4, L31: I recommend to move the definition of WT to the top of the introduction.	This has been adjusted
5. P5, L44: … the environment (i.e. WT) – I wonder, as technology is mentioned later in the sentence, whether the environment should be defined as the elderly care (facility) in this place?	This has been adjusted as it was incorrectly described. It now reads …interaction between the environment (i.e., WT) and the person influences the individuals' behaviour…
Methods<br>6. Please consider some information on research team and reflexivity: what kind of professional background or research focus do the researchers have?	This has been added.
7. P7, 42: The range of your targeted sample size is very broad. How do you determine the final size? How will you decide, whether 10 or 20 participants of each group will be the better fit? Will you discuss the concept of data saturation? You have a very broad approach which might make it difficult to identify saturation. Therefore, you might want to specify it here.	This has been adjusted as the aim is to interview 15 participants from each group of stakeholders.<br>The concept of saturation will not be discussed in accordance with the method chosen as the authors Braun and Clarke argue (Virginia Braun & Victoria Clarke (2021) To saturate or not to saturate? Questioning data saturation as a useful concept for thematic analysis and sample size rationales, Qualitative Research in Sport, Exercise and Health, 13:2, 201-216, DOI:10.1080/2159676X.2019.1704846)
8. P7, L43: … previous research: please add a reference.	This has been added
9. Please also add the topic guides for your interviews as a supplement.	This has been added
10. P8, L15: … using or making strategic decisions regarding WT …: please define, what kind of WT will be considered as inclusion criterion, and whether there are any exclusions in this regard. Please also define municipal elder care: is this inpatient / residential care or outpatient / home care, or both? Does municipal refer to the institution as being funded by municipality? Are privately funded institutions excluded? Maybe this concept does not even exist in Sweden, but it does in other countries, so an explanation to the international reader will be appreciated. Will you try to include all stakeholder groups within participating institutions, or will you recruit on the individual level?	There has been clarified that any WT used in municipality elder care is eligible for inclusion.<br>Municipal elder care in Sweden has been defined/described in the text<br><br>A clarification regarding that we try to involve stakeholders from all groups has been added.

11. P8, L44: ... new contacts will be established: in which way?	This has been clarified
12. P8, L44: ... to ensure a variety of collected data: what is your aim with regard to variety? Does it refer to the technologies involved?	This is described in the beginning of the data collection paragraph. *Organizations recruited to participate will be strategically selected to secure variation in municipal data, with different prerequisites that might influence the implementation of WT such as demography, size, economic conditions, numbers of older persons, etc*
13. P8, L47f: advisory board: I assume that the advisory board is already in place, as you are almost done with data collection? Then I would recommend to give more detailed information (profession, organisation or sector, number of participants) on your advisory board	The advisory board is still not fully recruited. Due to unforeseen situations (sickleave amongst the researchers) the data collection has been delayed as has the recruitment of advisory board.
14. P9, L42f: "The collected material will be analysed based on the respective publications that are planned": I should assume, that the research aim comes first and the publication second? I recommend to state the research aim and question for each participant group and the combined aim and question.	This sentence has been removed as the remainder of the text describes more in detail that each stakeholder's perspective as well as the different research questions will be analyzed.
15. P9, L55: When appropriate ... - This sounds slightly contradictive to what you state earlier as research questions and have in mind as final topics (safety etc, see your title). I am sure you will be easily able to clarify what your aim is, or with which methods you will analyse the different concepts you mention throughout your paper.	This has been adjusted as the main point was inform how the theoretical model (Competence–Press Model) will be used in the project.
16. P10, L25f: Data statement section: You should describe here what your plan for data sharing is. It is also not about access of your authors and team to data but about access of independent researchers and transparency of your research. If you do not plan to make raw data (=transcripts in this case) accessible, you should state it here and give a reason. I really recommend to read: "Recommendations for the Conduct, Reporting, Editing, and Publication of Scholarly Work in Medical Journals" – this is a brilliant paper that summarizes about everything that is helpful when publishing. See part III, letter L, number 2 on data sharing.	This has been added in line with other publications on the journal's homepage.

17. P10, L45: ... will be pseudonymized: in which way will participants receive a pseudonym and how will it be linked to personal data? Or could you also describe your method as anonymized?	The pseudonyms used will be with the stakeholders group (i.e. Older person, significant other, staff or decision-maker) and age as a label when using citations. When doing interviews the informant can never be anonymized to the researcher. This has been clarified
Discussion<br>18. P11, L31: ... safety, activity, ...: How will you ensure, that these concepts will be explored in your research? Will you explicitly incorporate these in your interview guides / questions? Or will you use them as predefined concepts in your analysis? Then you should explain it in the methods section.	The concepts are what WR is supposed to contribute to the user, we do not ask direct questions about these concepts (as you can see in the supplement) but ask about the informants' perception of what it is for. In of the interviews that have been conducted, these concepts come up when the questions have been asked in this way.
Minor comments (language, tables and figures, ...)<br>19. A general remark: please consider to use first person active voice, this is recommended by many journals.	Language revisions have been made
20. Overall your language is clear, there are some minor typos, please check your manuscript carefully before resubmission. I recommend to consider a native or C2-level English speaker to do a final check.	Language check has been made

Good luck!
Katharina Silies

Reviewer: 2
Dr. Rekha Elaswarapu, King's College London - Strand Campus
Comments to the Author:.

This is an interesting topic relevant to care of the elderly	Thank you for your support.
A couple of points to highlight are:<br>1. level of health related morbidity may influence perception of the use of WT;	This is of course something that influences the use and perception of WT. In the introduction we acknowledge that functional limitations appear due to natural aging could affect the perception, and this will of course be discussed in relation to older people in the forthcoming publications.
2. Familiarity and comfort with the use of WT could be a factor to consider so someone who is a technophobe may start from an entirely different point of view in their perspective of the use of WT.	This is also an interesting topic for several of the actor groups. In our Supplement Topic guide you might see that we intend to question regarding both use, purpose and introduction to welfare technology and thus

	we hope to be able to elaborate on that in future publications.

Reviewer: 3
Dr. Marie Elf, Dalarna University
Please see the attached document for any additional comments
Comments to the Author:
Comments on the manuscript "Do welfare technology contribute to safety, activity, participation, and independence within elder care? -a qualitative study protocol Journal"
bmjopen-2024-094424

Thanks for the opportunity to read and comment on your manuscript about welfare technology (WT). As you write, welfare technology is common and will play a pivotal role in elder care today, but it's not explored enough when it comes to how stakeholders perceive welfare technology and how it contributes to the individual's security, activity, participation, and independence. The manuscript is well written and easy to follow.	Thank you for your support!
My main concern is that I cannot clearly identify your specific contribution with the study. While WT has been studied extensively, it's important to highlight what makes your work unique or distinct within this broader field. Consider focusing on what new insights, methods, or perspectives you bring.	Thank you for the comment. This has been added to the end of the introduction to make this clearer.
You have not explained what kind of WT you will explore. Since WT is a central theme, the introduction should define what is meant by welfare technology, especially in the context of elder care. Implicitly I can understand that you mean high tech but that must be clear. WT doesn't have to refer to high-tech devices. It can mean a wide range of tools, including non-digital or aids such as wheelchairs, walkers, and other equipment to support the well-being, independence, and quality of life of individuals who may have physical or cognitive disabilities.<br>While welfare technology often suggests digital innovations like health-monitoring apps, smart home devices, or telehealth platforms, the term can also apply to more traditional assistive devices that serve the	Thank you for your comment. This is delicate as we have chosen to use the definition from the Nordic Welfare center that says *all technology* used to maintain or increase security, activity, participation or independence is considered to be welfare technology. So, this includes walkers etc.<br>In Sweden there is a narrower definition of welfare technology to embrace only digital technology that aims to contribute to security, activity, participation, and independence.<br>We have chosen not to give examples as we like the informants to describe what WT they have and what implications it gives to them. If asked for of course we mention different technologies, preferable technology we have perceived that they have f ex safety alarms if you see them have an alarm button.

same goals. You need to give examples of what kind of WT and devices you mean.	
In addition, you need to write more clearly what WT can contribute with when it comes to improved service, work environment, health etc...in what way I keep asking when reading the introduction.	Thank you for your comment. Some examples of what the benefit can be has been added.
You also need to more clearly discuss the role of WT in addressing safety, activity, participation, and independence. For example, how can WT (which one) enhance the safety of older adults? How might WT (which one) support physical activity? What role does technology play in helping older adults remain socially connected or engaged with their communities? How does welfare technology facilitate independence in daily living?	Thank you for your comment. This is what the project is about to explore from the perspective of the different stakeholders
Moreover, you write that the project can contribute knowledge on the implementation and consequences of WT for older persons, significant others, and staff, then the introduction should address implementation of WT and its challenges as a focus.	Thank you for your comment. This bullet point has been replaced in accordance with the instructions for the journal. It has also been clarified in the text that it is the perception and experiences that are in focus for the project and not the implementation and thus we have chosen not to write about implementation in the introduction.
What do you mean by implementation in the context of WT in elder care? This might include discussing what aspects of implementation are relevant, such as the processes, challenges, and necessary conditions for successfully integrating technology. How can implementation science explain the effectiveness and usability of WT for older persons, as well as its acceptance by staff and family members. Do you think implementation success differ among groups (older persons, significant others, and staff)?	We have tried to find where this is written in the manuscript so that we could give you an answer, but we can't find this wording. Also as pointed out in the previous answer we have tried to clarify that this project are foremost about the different stakeholders perceptions and experiences.
Without mentioned or explain further how WT can influence safety, independence etc... and implementation, the introduction feels incomplete or disconnected from the project's main objectives.	Some examples on what WT can contribute to ins added in the introduction.<br>Also regarding the concepts are what WR is supposed to contribute to the user, we do not ask direct questions about these concepts (as you can see in the supplement) but ask about the informants' perception of what it is for. In the interviews that have been conducted, these concepts come up when the questions have been asked in this way.
I wonder why your research questions do not mention safety, independence, participation	Thank you for the comment. This is a question we have been dealing with as it is

and implementation? I cannot see the connection to the title.	the definition of WT that consists of these terms but the terms are not defined in any way. We have concluded that we should not define them, but let our informants express how they view what WT means to them and what it gives them. Hopefully, these meanings can be linked to the different concepts.
You use safety and security interchangeable? Do you mean it is the same? For me they are slightly different concepts.	This has been adjusted as the term to be used should be security in accordance with the definition of welfare technology.
What questions will you ask the stakeholders? Can you give some examples?	A supplementary topic guide for the interviews has been added.
In which context are you planning to do your research? In elder care in normal housing or at residential care facilities? In several municipalities or in one? Could older adults have any disabilities? And what kind of WT? We need more information.	This has been clarified in the methods section.
Are you using any framework for reporting qualitative studies such as COREQ?	Yes, as stated on p 10 we will use the SRQR, Standards for Reporting Qualitative Research. This is equivalent with the COREQ.

Reviewer: 4
Dr. Kathleen Potter, Ryman Healthcare Ltd
Comments to the Author:

This paper describes a study protocol for a qualitative investigation of the thoughts and feelings of four different groups about the use of welfare technologies in the care of older people who require municipal care. The paper is generally well structured, clear and contains an appropriate level of detail. The study subject is important and relevant and I look forward to reading the authors' findings in due course.	
I have a few suggestions for improving the protocol description as noted below.<br>1. The paper would benefit from providing some more definitions of the terms used in a way that an international readership will understand. The terms that need definition early, including in the abstract preferably are:	Thank you for your comment, this has been adjusted as reported below.
a. Municipal elder care – this may be a specific European term or it may be a specific Swedish term, but it is not clear to	A definition/description of Swedish municipal elder care has been added.

this Australasian reader exactly what it means. Is it care provided in the home or is it care provided via an institution (a residential age care facility, for example) or is it both? Some clarification is required.	
b. Welfare technology – this term is defined in the second paragraph of the introduction, but it needs to be defined in the first paragraph I believe, so that the reader knows exactly what is being studied. Similarly, there needs to be a brief definition provided in the abstract. Some examples would also be useful, to help the reader understand the scope of the technologies being considered in the research.	The definition of welfare technology has been moved to the first paragraph. We have also added that WT in this project is a broader phenomenon embracing various technologies.
c. It would also be helpful to define how the researchers understand the terms 'security, activity, participation and independence" in the context of the demographic they are studying. A box might be useful, with all these terms defined, as the authors are applying them in their research, and this could be referred to by the reader, if the authors do not wish to include the definitions in the text of the paper.	Thank you for the comment. This is a question we have been dealing with as it is the definition of WT that consists of these terms but the terms are not defined in any way. We have concluded that we should not define them, but let our informants express how they view what WT means to them and what it gives them. Hopefully, these meanings can be linked to the different concepts.
2. This is a general comment, but the scope of the project seems very broad. On one had this is commendable, as the authors will capture a wide range of information about their subject, but on the other hand it does make me wonder how they will synthesize all this information into some coherent summary of the topic.	Thank you for your insightful comment.<br>Our intention is to describe for each group how they view<br>It is also very much about what answers we get and what it gives us in our analyses how we can proceed and synthesize the knowledge that is obtained.
3. A little more detail about how the authors will ensure an unbiased sampling of the population they are interested in interviewing would be useful. Using existing research networks is acceptable, but there is a danger of reinforcing an existing echo-chamber with only similar voices being heard by this approach. I would like to see more details about the efforts the authors have made to ensure their recruitment methods reach voices who are not always represented in this type of research. Will they interview immigrant care staff for instance, or only native Swedes?	Thank you for the comment. Wehave clarified that besides using our established networks that contacts through official contact channels will be made to ensure a variety of data. WE also elaborated a little bit more on the process where we ask operational managers for help with recruitment.<br>Regarding staff no exclusion is made more than to be able to perform an interview in Swedish. Thus, both native swedes and immigrant care staff will be interviewed.
4. There are a few clunky bits of English in the paper and a couple of simple spelling/grammatical errors. The authors	Thank you for the comment. The paper has been checked for grammar and spelling.

would benefit from asking a native English paper to read the draft and correct these minor errors. Two I picked up on were Page 7, line 3 'explains' should be 'explain' and Page 7, line 8 'difficulty to use' should be 'difficulty in using'. There were several others.	
Overall this is a great project and very timely and topical. The authors are to be commended on undertaking a challenging and interesting project and I look forward to reading their papers.	Thank you for your encouraging comment.

VERSION 2 - REVIEW

Reviewer 1

Name Silies, Katharina

Affiliation University of Lübeck, Institute for Social Medicine and Epidemiology, Nursing Research Unit

Date 19-Dec-2024

COI

Dear authors, I have been very pleased to have the opportunity to review your revised manuscript.

You addressed the considerable amount of reviewer comments comprehensively and improved your manuscript, from my point of view, successfully.

Minor comments:

1. Please check p 7 lines 17 – 20 for grammar errors.

2. Please check p 10 lines 46/47 (will be approached?)

3. Please check p 11 lines 42 and 49/50 for grammar errors.

I wish you all the best with your submission and your study. Indeed, this is an important topic and I hope you will have insightful results.

Have a well-earned Christmas holiday 😊

Katharina Silies

Reviewer 3

Name **Elf, Marie**

Affiliation **Dalarna University, School of education, health and social studies**

Date **09-Jan-2025**

COI

Comments on the revised manuscript: bmjopen-2024-094424.R1

Title: Does welfare technology contribute to security, activity, participation, and independence within elder care? - A qualitative study protocol.

Thank you for your thorough responses and the revisions where needed. I have no further comments. Good work, and it will be lovely to follow.

VERSION 2 - AUTHOR RESPONSE

Reviewer: 1
Dr. Katharina Silies, University of Lübeck
Comments to the Author:
Dear authors, I have been very pleased to have the opportunity to review your revised manuscript.
You addressed the considerable amount of reviewer comments comprehensively and improved your manuscript, from my point of view, successfully.

Thank you for your kind response,

Minor comments:
1. Please check p 7 lines 17 – 20 for grammar errors.

This has been corrected

2. Please check p 10 lines 46/47 (will be approached?)

According to our languagesupport the use of the word employed is more suitable in the context so we have keept it.

3. Please check p 11 lines 42 and 49/50 for grammar errors.

This has been rewritten.

I wish you all the best with your submission and your study. Indeed, this is an important topic and I hope you will have insightful results.
Have a well-earned Christmas holiday [illegible]

Katharina Silies

Reviewer: 3
Dr. Marie Elf, Dalarna University
Comments to the Author:
Comments on the revised manuscript: bmjopen-2024-094424.R1
Title: Does welfare technology contribute to security, activity, participation, and independence within elder care? - A qualitative study protocol.
Thank you for your thorough responses and the revisions where needed. I have no further comments. Good work, and it will be lovely to follow.

Thank you for your nice answer.
